# Prostaglandin production in mouse mammary tumour cells confers invasive growth potential by inducing hepatocyte growth factor in stromal fibroblasts

**DOI:** 10.1038/sj.bjc.6690677

**Published:** 1999-09

**Authors:** N Matsumoto-Taniura, K Matsumoto, T Nakamura

**Affiliations:** Division of Biochemistry, Department of Oncology, Biomedical Research Centre, Osaka University Medical School, Suita, Osaka, 565-0871, Japan

**Keywords:** HGF, prostaglandin E_2_, tumour invasion, tumour–stromal interaction, mammary tumour

## Abstract

Interactions between stromal and mammary tumour cells play a crucial role in determining the malignant behaviour of tumour cells. Although MMT mouse mammary tumour cells do not produce hepatocyte growth factor (HGF), addition of conditioned medium (CM) from MMT cells to cultures of human fibroblasts derived from skin and breast tissues stimulated the production of HGF, thereby indicating that MMT cells secrete an inducing factor for HGF. This HGF-inducing factor, purified from MMT-derived CM, proved to be prostaglandin E_2_ (PGE_2_). Consistently, treatment of MMT cells with indomethacin, an inhibitor of cyclooxygenase, abolished this HGF-inducing activity in MMT-derived CM, while treatment of MMT cells with HGF stimulated cell growth and cell motility. Likewise, HGF strongly enhanced urokinase-type plasminogen activator activity and invasion of MMT cells through Matrigel: a 15-fold stimulation in the invasion of MMT cells was seen by HGF. Finally, MMT cells in the upper compartment were co-cultivated with fibroblasts in the lower compartment of the Matrigel chamber, HGF levels in the co-culture system exceeded the level in fibroblasts alone and suppression occurred with exposure to indomethacin. Together with increase in the HGF level, the invasion of MMT cells was enhanced by co-cultivation with fibroblasts, whereas the increased invasion of MMT cells was significantly inhibited by an anti-HGF antibody and by indomethacin. These results indicate mutual interactions between MMT cells and fibroblasts: MMT-derived PGE_2_ plays a role in up-regulating HGF production in fibroblasts, while fibroblast-derived HGF leads to invasive growth in MMT cells. The mutual interactions mediated by HGF and prostaglandins may possibly be a mechanism regulating malignant behaviour of mammary tumour cells, through tumour–stromal interactions. © 1999 Cancer Research Campaign


*British Journal of Cancer* (1999) **81**(2), 194–202
© 1999 Cancer Research Campaign
Article no. bjoc.1999.0677

### Prostaglandin production in mouse mammary tumour cells confers invasive growth potential by inducing hepatocyte growth factor in stromal fibroblasts

**N Matsumoto-Taniura, K Matsumoto and T Nakamura**

Division of Biochemistry, Department of Oncology, Biomedical Research Centre, Osaka University Medical School, Suita, Osaka 565-0871, Japan

**Summary** Interactions between stromal and mammary tumour cells play a crucial role in determining the malignant behaviour of tumour cells. Although MMT mouse mammary tumour cells do not produce hepatocyte growth factor (HGF), addition of conditioned medium (CM) from MMT cells to cultures of human fibroblasts derived from skin and breast tissues stimulated the production of HGF, thereby indicating that MMT cells secrete an inducing factor for HGF. This HGF-inducing factor, purified from MMT-derived CM, proved to be prostaglandin $E_2$ ($PGE_2$). Consistently, treatment of MMT cells with indomethacin, an inhibitor of cyclooxygenase, abolished this HGF-inducing activity in MMT-derived CM, while treatment of MMT cells with HGF stimulated cell growth and cell motility. Likewise, HGF strongly enhanced urokinase-type plasminogen activator activity and invasion of MMT cells through Matrigel: a 15-fold stimulation in the invasion of MMT cells was seen by HGF. Finally, MMT cells in the upper compartment were co-cultivated with fibroblasts in the lower compartment of the Matrigel chamber, HGF levels in the co-culture system exceeded the level in fibroblasts alone and suppression occurred with exposure to indomethacin. Together with increase in the HGF level, the invasion of MMT cells was enhanced by co-cultivation with fibroblasts, whereas the increased invasion of MMT cells was significantly inhibited by an anti-HGF antibody and by indomethacin. These results indicate mutual interactions between MMT cells and fibroblasts: MMT-derived $PGE_2$ plays a role in up-regulating HGF production in fibroblasts, while fibroblast-derived HGF leads to invasive growth in MMT cells. The mutual interactions mediated by HGF and prostaglandins may possibly be a mechanism regulating malignant behaviour of mammary tumour cells, through tumour–stromal interactions.

© 1999 Cancer Research Campaign

**Keywords**: HGF; prostaglandin $E_2$; tumour invasion; tumour–stromal interaction, mammary tumour

Interactions between epithelium and mesenchyme (or stroma) mediate crucial aspects of normal development, tissue morphogenesis and neoplasia. In tissue recombination, growth, differentiation and morphogenesis of developing epithelia are regulated either inductively or permissively by neighbouring mesenchyme, including pancreas, salivary gland, kidney, mammary gland, etc. (Grobstein, 1967; Sakakura, 1991; Birchmeier and Birchmeier, 1993). Host stromal influence on epithelial neoplasia and malignant progression of carcinoma cells have also been noted in various tumours, including cancers in prostate, stomach, skin, oral cavity, mammary gland, etc. (van den Hoof, 1988). In vivo growth of certain carcinoma cells was markedly accelerated by a broad spectrum of fibroblasts, and in vitro invasiveness of several carcinoma cells was induced by co-cultivation with stromal fibroblasts (Picard et al, 1986; Grey et al, 1989; Camps et al, 1990; Matsumoto et al, 1994). Although matrix metalloproteinases, growth factors and cell motility factors are implicated in tumour–stromal interactions, less is known of molecular mechanisms which confer invasive growth of cancer cells through interactions with surrounding stroma.

Hepatocyte growth factor (HGF), originally identified as a potent mitogen for hepatocytes (Nakamura et al, 1984), is a kringle-containing growth factor which has mitogenic, motogenic and morphogenic activities in a wide variety of cells (Nakamura et al, 1989; Montesano et al, 1991; Zarnegar and Michalopoulos, 1995; Matsumoto and Nakamura, 1997). HGF specifically activates the Met/HGF receptor of heterodimeric tyrosine kinase, which is expressed on a wide variety of epithelial cells, endothelial cells and several mesenchymal cells. During normal development, HGF supports growth, migration and morphogenesis of organs and tissues, including the liver, kidney, tooth, limb muscle, lung and mammary gland, as a mesenchymal-derived paracrine factor (Santos et al, 1994; Bladt et al, 1995; Niranjan et al, 1995; Soriano et al, 1995; Yang et al, 1995; Tabata et al, 1996; Ohmichi et al, 1998). Likewise, HGF plays a ‘trophic’ role to enhance regeneration of organs, including the liver, kidney and lung, as a stromal-derived factor (see review, Matsumoto and Nakamura, 1997). Thus, HGF seems to be a mediator in epithelial–mesenchymal (or –stromal) interactions during tissue formation and repair.

Accumulating evidence shows that HGF is likely to play a role in tumour progression through tumour and host stromal interactions (Seslar et al, 1993; Rosen et al, 1994; Matsumoto et al, 1996; Nakamura et al, 1997). As scatter factor, originally identified as fibroblast-derived cell motility factor for epithelial cells (Stoker et al, 1987), proved to be identical with HGF (Furlong et al, 1991; Konishi et al, 1991; Weidner et al, 1991), HGF potently stimulates migration and invasion of various types of cells, including carcinoma cells (Weidner et al, 1990; Jiang et al, 1993; Matsumoto et al, 1994, 1996; Nakamura et al, 1997), and autocrine activation of Met results in increased tumourigenicity and metastasis

*Received 10 November 1998*
*Revised 9 March 1999*
*Accepted 11 March 1999*

*Correspondence to:* T Nakamura

(Bellusci et al, 1994; Jeffers et al, 1996). HGF is an angiogenic factor and plays a role in tumour angiogenesis (Bussolino et al, 1992; Grant et al, 1993; Lamszus et al, 1997). Fibroblast-derived factor, which induces in vitro invasion of carcinoma cells, proved to be HGF (Matsumoto et al, 1994). On the other hand, various types of carcinoma cells secrete soluble factors that regulate the production of HGF in stromal fibroblasts (Seslar et al, 1993; Matsumoto et al, 1996; Nakamura et al, 1997). These results suggest the presence of the mutual interaction between carcinoma cells and fibroblasts, as mediated by tumour-derived regulator for expression of HGF and fibroblast-derived HGF, which affects invasive growth of tumour cells.

During a search for tumour-derived inducers for expression of HGF in fibroblasts, we found that the murine mammary carcinoma cell line, MMT cells, secreted a distinct type of HGF-inducer for fibroblasts. We now report that the MMT-derived HGF-inducer is prostaglandin $E_2$ ($PGE_2$), and the biological significance of $PGE_2$ in invasive growth of MMT cells was investigated.

### MATERIALS AND METHODS

### Cell culture

MMT mouse mammary carcinoma cells were obtained from the Human Science Research Resources Bank, Japan. MMT cell line was originally established from a spontaneous mammary tumour which arose in a (C57BL × Af)$F_1$ female mouse (Sykes et al, 1968). MMT cells are epithelial in appearance and produce mouse mammary tumour virus, implicating that MMT cells were originally transformed by mammary tumour virus infection (Sykes et al, 1968). MMT cells were cultured in modified Eagle's medium (MEM) supplemented with 10% calf serum. Normal or tumour associated human fibroblasts were, respectively, proliferated outward from the skin and breast cancer tissues obtained during surgery and the cells were cultured in Dulbecco's modified Eagle's medium (DMEM) supplemented with 10% fetal calf serum (FCS).

### Growth factors and antibodies

Human recombinant HGF was purified from culture medium of Chinese hamster ovary cells transfected with expression plasmid for human HGF cDNA (Nakamura et al, 1989; Seki et al, 1990). The purity of HGF exceeded 98%, as determined by sodium dodecyl sulphate polyacrylamide gel electrophoresis (SDS-PAGE) and protein staining. Human recombinant platelet-derived growth factor (PDGF) and bovine recombinant basic fibroblast growth factor (bFGF) were obtained from Toyobo Co. (Osaka, Japan). Human recombinant interleukin-1α (IL-1α) and IL-1β were obtained from Genzyme Co. (Boston, MA, USA). A recombinant IL-1 receptor antagonist was obtained from R&D systems Co. (Minneapolis, MN, USA) and monoclonal anti-human epidermal growth factor (EGF) receptor antibody were obtained from Genzyme Co. (Boston, MA, USA). Polyclonal anti-PDGF antibody was obtained from Promega (Madison, WI, USA) and monoclonal anti-bFGF (Matsuzaki et al, 1989) was a kind gift from Dr K Nishikawa (Kanazawa Medical College). Polyclonal antibody against human HGF was prepared from the serum of a rabbit immunized with human recombinant HGF. IgG was purified using protein A-Sepharose (Pharmacia, Uppsala, Sweden) and anti-human HGF IgG (1 μg $ml^{-1}$) completely neutralized biological activities of 1 ng $ml^{-1}$ human HGF.

### Measurement of cell growth and scattering

To measure cell growth, MMT cells were seeded on 12-well plates (Costar, Cambridge, MA, USA) at a density of $1.1 \times 10^3$ cells $cm^{-2}$ and cultured for 24 h. After washing with MEM, the cells were cultured for 4.5 days in serum-free MEM in the absence or presence of HGF. The medium was changed on day 3.

For the measurement of cell scattering, MMT cells were seeded on 24-well plates (Costar, Cambridge, MA, USA) at a density of $2.8 \times 10^3$ cells $cm^{-2}$ and cultured for 48 h. After medium was changed, HGF was added and the cells were cultured for 18 h.

### In-vitro invasion assay

In-vitro invasion of MMT cells was measured using a Matrigel invasion chamber (Collaborative Biomedical Products, Bedford, MA, USA). MMT cells were seeded onto the upper compartment of a Matrigel chamber at a density of $6.7 \times 10^4$ cells $cm^{-2}$ and cultured in MEM containing 10% FCS. HGF was added to the lower compartment and cells were cultured for 15 h. Invasive cells penetrating through Matrigel components and pores to the underside of the membrane were stained with haematoxylin and eosin, viewed microscopically and counted.

For co-cultivation of MMT cells and fibroblasts, human dermal fibroblasts were initially plated onto the lower compartment of a Matrigel invasion chamber, at a density of $5 \times 10^4$ cells $cm^{-2}$ and cultured for 24 h. The medium was changed to fresh medium composed of MEM/DMEM (1/1) containing 5% FCS and 5% calf serum. The MMT cells were seeded onto the upper compartment at a density of $6.7 \times 10^4$ cells $cm^{-2}$. The cells were cultured for 48 h in the absence or presence of preimmune IgG, anti-human HGF IgG (10 μg $ml^{-1}$), $10^{-7}$ M indomethacin or $10^{-7}$ M indomethacin plus $10^{-6}$ M $PGE_2$.

### Measurement of HGF production in fibroblasts

Human fibroblasts were seeded on a 48-well plate (Costar, Cambridge, MA, USA) at a density of $5 \times 10^4$ cells $cm^{-2}$ and cultured for 24 h. After replacing the medium with DMEM supplemented with 1% FCS and 2 μg $ml^{-1}$ heparin, test samples were added to each well. Following a 24 h culture, the concentration of HGF in the medium was measured using enzyme-linked immunosorbent assay (ELISA), as described elsewhere (Matsumoto et al, 1996).

### Measurement of $PGE_2$ production in MMT cells

Subconfluent MMT cells were washed twice with serum-free MEM, cultured in serum-free MEM, with or without indomethacin for 24 h and the conditioned medium (CM) was collected. The concentration of $PGE_2$ in the medium was determined using an ELISA kit obtained from Cayman Co. (Ann Arbor, MI, USA). This ELISA specifically detects $PGE_2$, not other prostaglandins.

### Zymographies for gelatinase and urokinase-type plasminogen activator

Subconfluent MMT cells were cultured in serum-free MEM, with or without HGF for 24 h, and the CM was collected. For the measurement of gelatinase activity, samples were subjected to SDS-PAGE, using a 10% polyacrylamide gel containing 1 mg $ml^{-1}$

© 1999 Cancer Research Campaign

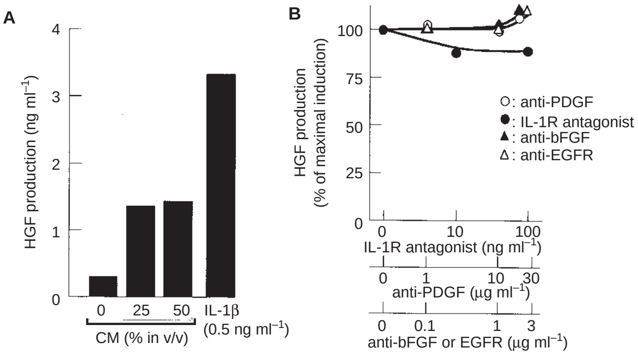

**Figure 1** Stimulatory effect of conditioned medium (CM) from MMT cells on HGF production in human skin fibroblasts. (**A**) Stimulation of HGF production by MMT-derived CM. (**B**) Effects of antibodies and an antagonist against polypeptide HGF-inducers. Fibroblasts were seeded on 48-well plates and cultured for 24 h. After replacing the medium with fresh DMEM supplemented with 1% FCS and 2 µg ml$^{-1}$ heparin, CM from MMT cells was added in the absence or presence of antibodies and an antagonist. The cells were cultured for 24 h and the concentration of HGF in the medium was measured by ELISA. Values represent the mean of triplicate measurements

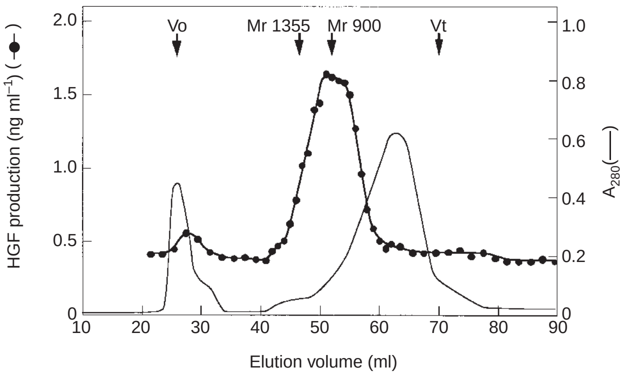

**Figure 2** Elution profile of HGF-inducing activity from MMT-derived CM in molecular sieve chromatography with a Sephadex G-25 column. The CM was concentrated by lyophilization and applied to a Sephadex G-25 column equilibrated with 15 mM phosphate buffer (pH 7.2), 0.15 M sodium chloride. Eluted fractions were subjected to assay for HGF-inducing activity, using human skin fibroblasts gelatin. After electrophoresis, the gel was washed in 2.5% Triton X-100, and incubated in 50 mM Tris–HCl buffer (pH 8.0) containing 0.5 mM calcium chloride and 1 mM zinc chloride for 24 h at 37°C. The gel was stained with Coomassie brilliant blue and photographed. For the measurement of urokinase-type plasminogen activator (u-PA), culture supernatants were prepared and analysed as described elsewhere (Vassalli et al, 1984).

### Purification of MMT-derived HGF-inducer

After MMT cells grew to confluency, the culture medium was changed to serum-free medium and the cells were cultured for a further 48 h. CM was concentrated by lyophilization and subjected to molecular sieve chromatography, using a Sephadex G-25 column (Pharmacia Biotech, Uppsala, Sweden), equilibrated with 15 mM phosphate buffer (pH 7.2), 0.15 M sodium chloride. Active fractions were pooled, concentrated by lyophilization, and applied to reverse phase high-performance liquid chromatography (RP-HPLC) with a $C_{18}$ column. Absorbed materials were eluted by a concentration gradient of acetonitrile. The eluate was concentrated by vacuum centrifugation and dissolved in $H_2O$.

### RESULTS

### Characterization of MMT-derived HGF-inducer

HGF-inducing activity was determined by measuring HGF production in cultures of human skin fibroblasts after addition of CM from MMT mouse mammary tumour cells. The addition of MMT-derived CM stimulated HGF production in cultures of fibroblasts (Figure 1A): a four- to fivefold increase in HGF level in fibroblast cultures occurred when CM was added at 50% (v/v), whereas its potential to stimulate HGF-production was less than that of 0.5 ng ml$^{-1}$ IL-1β. Since the MMT-derived CM itself did not contain a detectable level of HGF (not shown), our observations indicated that MMT cells secrete a factor which stimulates the production of HGF in normal human fibroblasts.

Our previous studies demonstrated that several types of tumour cells secrete inducing factors for HGF production in fibroblasts, and these factors were identified to be IL-1α, IL-1β, PDGF and bFGF (Matsumoto et al, 1996; Nakamura et al, 1997). We therefore asked whether the MMT-derived HGF-inducer is identical to one of these factors. MMT-derived CM was added to fibroblast cultures and the cells were cultured in the absence or presence of an IL-1 receptor (IL-1R) antagonist or an antibody against PDGF, bFGF or EGF-R. IL-1R antagonist and these antibodies almost completely inhibited the respective activities of IL-1α, IL-1β, PDGF, bFGF and TGF-α to stimulate HGF production (not shown). The stimulatory effect of MMT-derived CM on HGF production was slightly inhibited by the IL-1R antagonist but was hardly inhibited by any of the antibodies (Figure 1B). This indicates that the HGF-inducer derived from MMT cells is distinct from these cytokines and growth factors.

MMT-derived CM was concentrated by lyophilization, subjected to molecular sieve chromatography, using a Sephadex G-25 column, and the eluted fractions were tested to determine if they would stimulate HGF production in fibroblast cultures (Figure 2). HGF-inducing activity was eluted as two distinct peaks: the major peak eluted at positions of $M_r$ around 900 and the minor peak eluted near $V_o$ position.

### Identification of MMT-derived HGF inducer

We repeated molecular sieve chromatography and active fractions from CM were pooled and lyophilized. The pooled material was dissolved in $H_2O$, dialysed against $H_2O$, and subjected to RP-HPLC with a $C_{18}$ column (Figure 3A). The adsorbed materials were eluted with a concentration gradient of acetonitrile and the eluted fractions were subjected to assay. When the pooled material from CM of MMT cells was applied to RP-HPLC, HGF-inducing activity was seen as a single peak of fraction 55. It is worth noting that the HGF-inducing activity eluted after RP-HPLC was comparable to that seen with 0.5 ng ml$^{-1}$ IL-1β (a dose which gives the maximal activity of IL-1β) and it was approximately twofold higher than that of the maximal activity seen in the pooled materials subjected to RP-HPLC. The potentiation of HGF-inducing activity after RP-HPLC means that an inhibitory factor which suppresses HGF-inducing factor in MMT-derived CM was dissociated during RP-HPLC.

 © 1999 Cancer Research Campaign

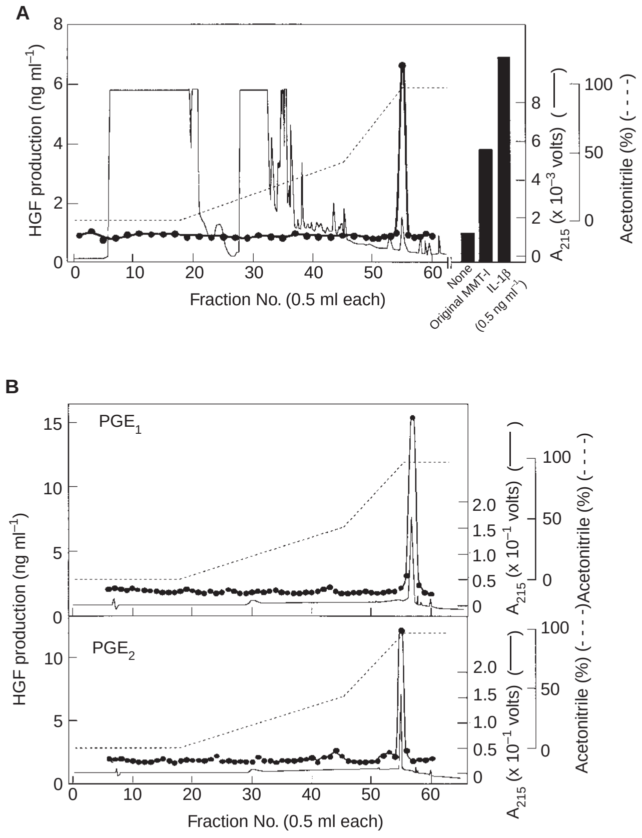

**Figure 3** Elution profile of HGF-inducing activity from MMT-derived CM (**A**) and $PGE_1$ and $PGE_2$ (**B**) in $C_{18}$ RP-HPLC. Pooled active fractions after molecular sieve chromatography were concentrated and subjected to $C_{18}$ RP-HPLC. The absorbed materials were eluted with a concentration gradient of acetonitrile and eluted fractions were subjected to assay for HGF-inducing activity

The above results indicated that the MMT-derived HGF-inducer was a lipophilic molecule with a relatively low $M_r$, and we speculated it might be prostaglandins. We therefore tested whether various prostaglandins might stimulate HGF production in the fibroblasts. Among prostaglandins tested, including prostaglandin $D_2$ ($PGD_2$), $PGE_1$, $PGE_2$, $PGF_{2\alpha}$, $PGI_2$, staurosporin $A_2$ and thromboxan $B_2$, $PGE_1$ and $PGE_2$ patently stimulated HGF production in the fibroblasts (not shown). We therefore applied authentic $PGE_1$ and $PGE_2$, respectively, to a $C_{18}$ RP-HPLC column followed by elution under the same conditions as used for MMT-derived HGF-inducer (Figure 3B). $PGE_1$ eluted as a major single peak at fraction 57 and HGF-inducing activity in the elute coincided with the peak, while $PGE_2$ eluted at fraction 55, thus, coinciding with its biological activity to stimulate HGF production. Taken together, these results strongly suggest that the HGF-inducer secreted from MMT cells is $PGE_2$.

To obtain further evidence that the HGF-inducer derived from MMT cells is $PGE_2$, MMT cells were cultured in the presence of indomethacin, a specific inhibitor for cyclooxygenase, and HGF-inducing activity in the CM was measured. HGF-inducing activity in CM from MMT cells was almost completely inhibited by exposure to indomethacin, in a dose-dependent manner (Figure 4A). Likewise, HGF-inducing activity in CM of MMT cells treated

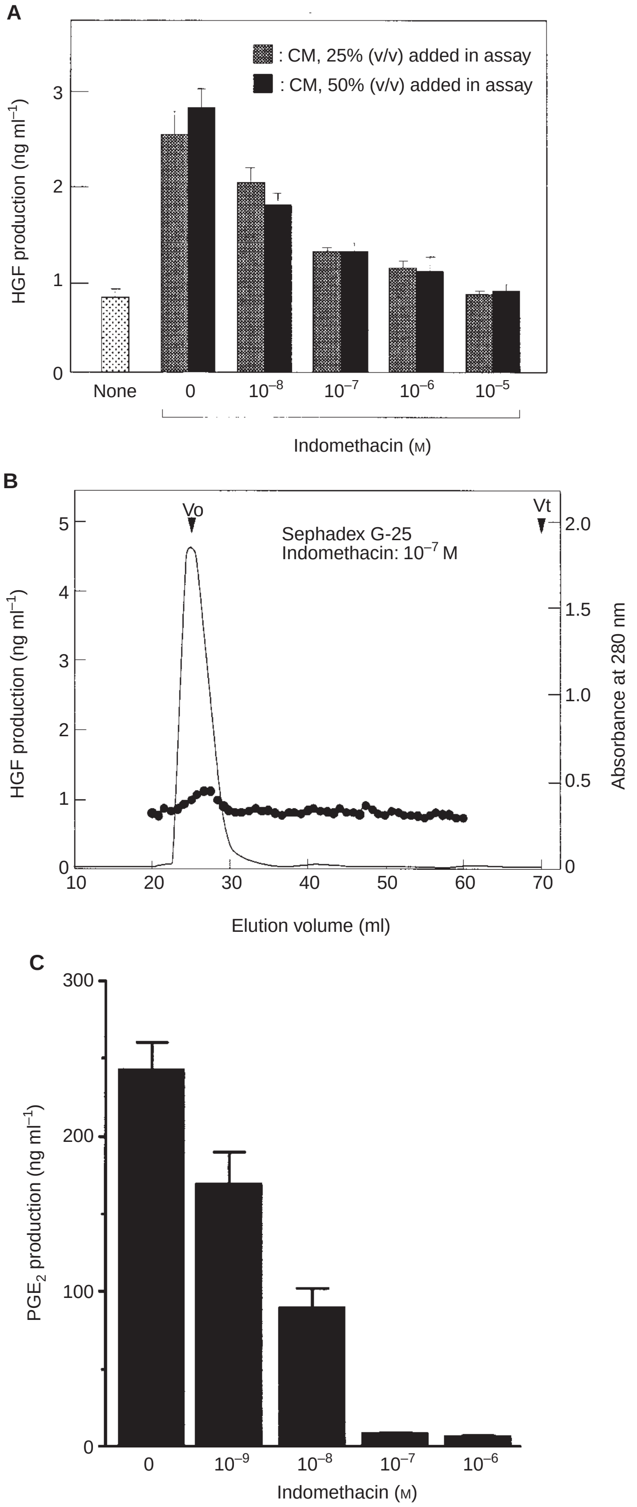

**Figure 4** Inhibition of HGF-inducing activity in CM from MMT cells exposed to indomethacin (**A, B**) and $PGE_2$ production in MMT cells (**C**). (**A**) Concentration-dependent inhibition of HGF-inducing activity in MMT-derived CM by indomethacin. (**B**) Elution profile of HGF-inducing activity in CM of MMT cells exposed or not exposed to $10^{-7}$ M indomethacin. Subconfluent MMT cells were cultured in the absence or presence of indomethacin for 48 h and CM from indomethacin-exposed MMT cells were subjected to assay for HGF-inducing activity (**A**) or molecular sieve chromatography with Sephadex G-25 column (**B**). (**C**) $PGE_2$ production in MMT cells cultured with or without indomethacin for 24 h. $PGE_2$ production in CM of MMT cells was measured using ELISA

© 1999 Cancer Research Campaign 

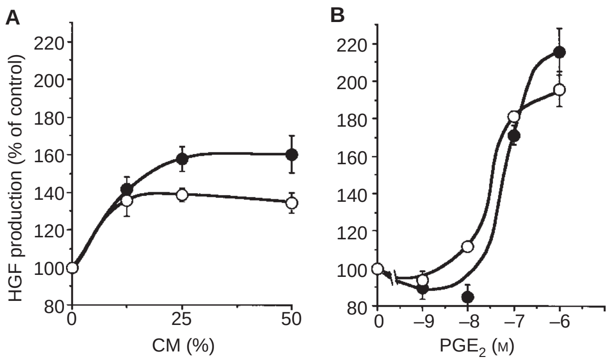

**Figure 5** Stimulatory effects of MMT-derived CM (**A**) and $PGE_2$ (**B**) on HGF production in human fibroblasts derived from normal breast and breast carcinoma tissues. Values represent the mean ± s.d. of triplicate measurements

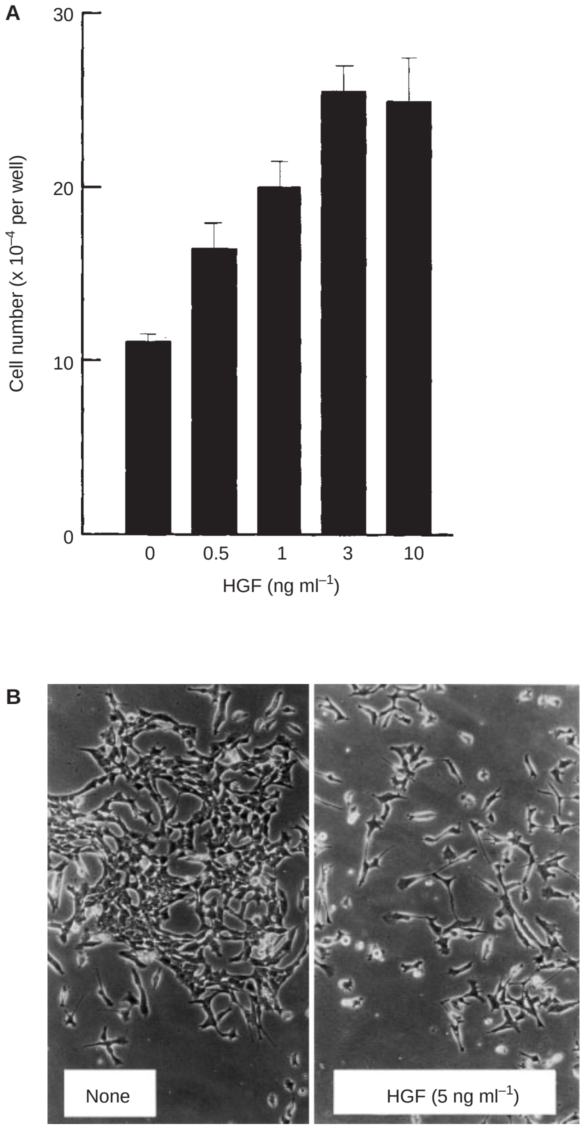

**Figure 6** Enhancement of cell growth (**A**) and cell scattering (**B**) of MMT cells by HGF. For measurement of cell growth, MMT cells were cultured for 4.5 days in serum-free MEM in the absence or presence of HGF. For the measurement of cell scattering, MMT cells were cultured in the absence or presence of HGF for 18 h

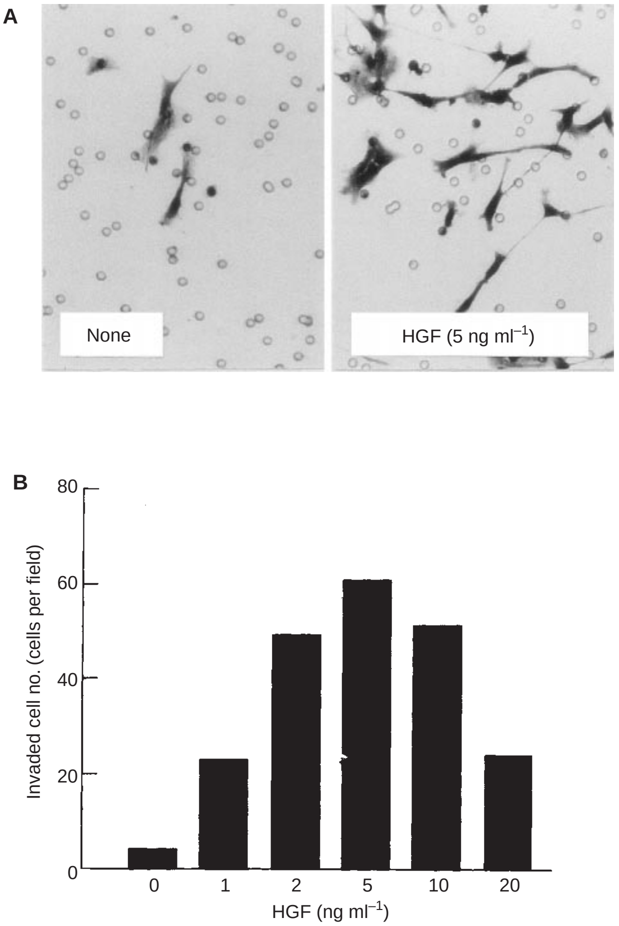

**Figure 7** Stimulatory effect of HGF on in vitro invasion of MMT cells. (**A**) Appearance of invasive cells migrating under the membrane. (**B**) Dose-dependent enhancement of invasion of MMT cells by HGF. MMT cells were seeded on Matrigel basement membrane components for 2 days in the absence or presence of HGF. Invasive cells invading through Matrigel and 8-μm pores of the filter membrane to the underside of the membrane were stained with haematoxylin and counted, as viewed microscopically

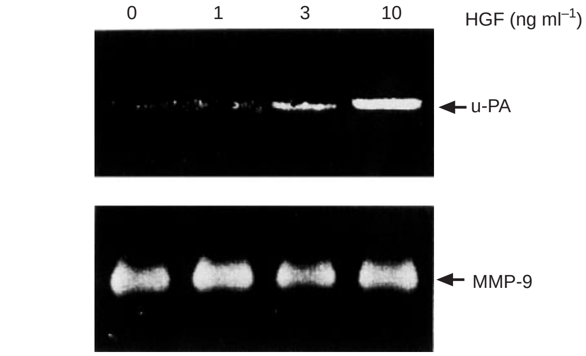

**Figure 8** Regulation of u-PA and gelatinase activities in MMT cells by HGF. u-PA activity (**A**) and gelatinase activity (**B**) were measured in CM from MMT cells. MMT cells were cultured for 24 h in the absence or presence of HGF, and CM was subjected to SDS-PAGE. u-PA and gelatinase activities were respectively measured using zymographical methods

© 1999 Cancer Research Campaign

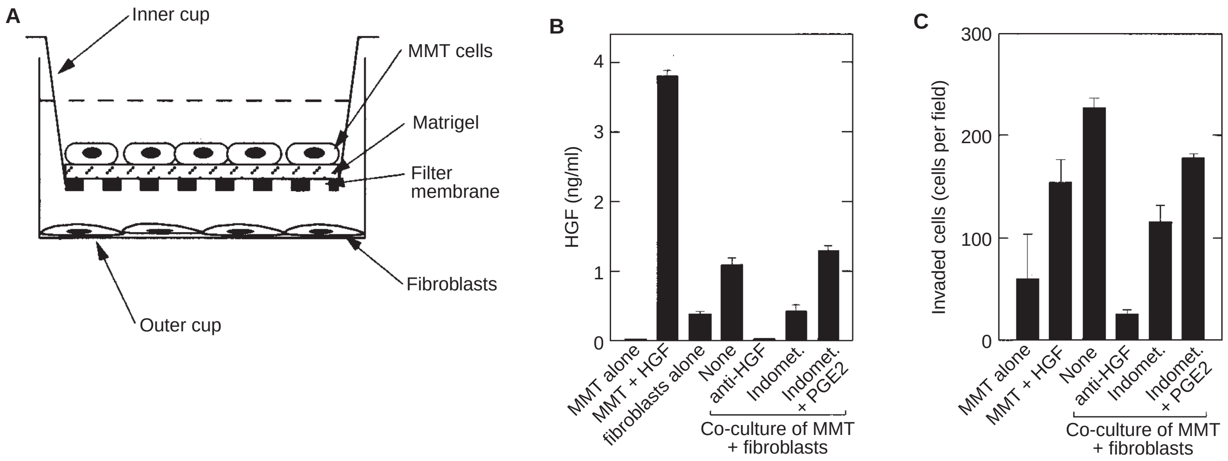

**Figure 9** In vitro invasion of MMT cells cocultured with fibroblasts. (**A**) Diagram showing method we used to co-culture MMT cells and fibroblasts. (**B**) HGF concentration in co-culture system of MMT cells and fibroblasts. (**C**) Number of invasive MMT cells in the co-culture of MMT cells and fibroblasts. Human fibroblasts were initially seeded on 24-well plates, and MMT cells were seeded in the upper compartment of a 24-well Matrigel invasion chamber. Cells were cultured for 48 h in the absence or presence of 5 ng $ml^{-1}$ HGF, 10 μg $ml^{-1}$ anti-HGF IgG, $10^{-7}$ M indomethacin, or $10^{-7}$ M indomethacin plus $10^{-6}$ M $PGE_2$, and the number of invasive cells was enumerated with $10^{-7}$ M indomethacin was measured after molecular sieve chromatography with Sephadex G-25 (Figure 4B). The elution profile clearly indicated that the HGF-inducing activity in the major peak corresponding to $M_r$ around 900 Da was almost completely diminished, whereas the HGF-inducing activity in the minor peak which eluted near $V_o$ position was not inhibited. We next measured $PGE_2$ production by MMT cells, using a specific ELISA (Figure 4C). During 24-h culture, $PGE_2$ level in the CM of MMT cells reached 242 ng $ml^{-1}$ ($6.9 \times 10^{-7}$ M). Furthermore, the addition of indomethacin dose-dependently inhibited $PGE_2$ production in MMT cells and $PGE_2$ production in the cells treated with $10^{-7}$ M indomethacin was only 1/30 of that seen in control culture. Together with these results, we concluded that the HGF-inducer from MMT cells was $PGE_2$.

Since the MMT cells originated from mammary tissue, we asked if HGF production in human fibroblasts derived from normal breast and breast carcinoma tissues in a patient with breast carcinoma was regulated by MMT-derived CM and $PGE_2$ (Figure 5). The basal HGF production in fibroblasts from normal and breast carcinoma tissues was $3.74 \pm 0.12$ ng $ml^{-1}$ and $5.27 \pm 0.14$ ng $ml^{-1}$ respectively. Although HGF production in fibroblasts from breast carcinoma tissue was higher than that in normal breast fibroblasts, HGF production in fibroblasts from breast carcinoma tissue was not consistently higher than that in normal breast fibroblasts to some extent, it was not consistent (not shown). HGF production in breast fibroblasts from both normal and tumour tissues was dose-dependently stimulated by both MMT-derived CM (Figure 5A) and $PGE_2$ (Figure 5B). Similar stimulatory effects were seen in other human breast fibroblasts derived from other patients with breast cancer (not shown).

### Biological effects of HGF on MMT cells

On the basis of our data that MMT cells secrete $PGE_2$ as a potent HGF inducer for fibroblasts, we assume that MMT cells might stimulate HGF production in fibroblasts and fibroblast-derived HGF might affect growth, migration and invasion of MMT cells. Interactions of tumour cells with stromal fibroblasts have a significant effect on the malignant behaviour of carcinoma cells (Lippman and Dickson, 1990; Sakakura, 1991; Wernert, 1997). When HGF was added to cultures of MMT cells, HGF stimulated proliferation of the cells, in a dose-dependent manner (Figure 6A). The maximal activity with a 2.5-fold increase in cell number was seen with 3–10 ng $ml^{-1}$ HGF. In addition to cell growth stimulation, HGF stimulated migration of the cells. Addition of HGF in MMT cells in monolayer culture induced scattering of the cells (Figure 6B), thereby indicating that HGF stimulates motility of MMT cells.

To determine if HGF would affect invasion of MMT cells, the cells were cultured in a Matrigel invasion chamber in the absence or presence of HGF (Figure 7A). Although some cells invaded through the Matrigel basement membrane components and migrated under the filter membrane in the absence of HGF, the addition of HGF strongly enhanced invasion of these cells. The maximal effect by 15-fold stimulation was seen with 5 ng $ml^{-1}$ HGF, while at a higher concentration, the number of invading cells decreased (Figure 7B). Therefore, HGF may stimulate the invasion of MMT cells through the basement membrane.

Since the process of invasion requires increased activities in both cell migration and extracellular proteolysis, we investigated the effects of HGF on extracellular protease activities in MMT cells. HGF-treatment strongly increased u-PA activity, in a dose-dependent manner (Figure 8). On the other hand, although MMT cells secreted 92 kDa gelatinase (MMP-9), this secretion remained unchanged with HGF-treatment (Figure 8).

### In vitro invasion through tumour–stromal interaction

Based on findings that MMT cells secrete $PGE_2$ as an inducer for HGF production in fibroblasts, while HGF strongly stimulates invasion of MMT cells, we set up a co-culture method, using a Matrigel invasion chamber in which fibroblasts were cultured in the lower compartment and MMT cells were cultured in the upper compartment (Figure 9A). This co-culture system mimics the

© 1999 Cancer Research Campaign 

tumour–stromal interaction through basement membrane components. Without co-cultured MMT cells, basal HGF production in fibroblasts was 0.4 ng ml$^{-1}$ during 48-h culture, but the production of HGF was stimulated to 1.1 ng ml$^{-1}$ by co-cultivation with MMT cells (Figure 9B). HGF was undetectable when anti-HGF IgG was added to this culture. The addition of $10^{-7}$ M indomethacin decreased the HGF to near basal levels seen in the culture of fibroblasts alone, indicating that the increase in HGF level under this co-culture condition was probably due to $PGE_2$ secreted from MMT cells. Simultaneous addition of $10^{-6}$ M $PGE_2$ and $10^{-7}$ M indomethacin to this culture again increased HGF levels 3.1-fold higher than seen in the culture with $10^{-7}$ M indomethacin alone.

When MMT cells were cultured alone, the addition of 5 ng ml$^{-1}$ HGF stimulated the invasion of MMT cells to a 2.6-fold higher level than seen without HGF (Figure 9C). The addition of 10 μg ml$^{-1}$ anti-HGF IgG almost completely neutralized the invasion of MMT cells stimulated by 5 ng ml$^{-1}$ HGF, while anti-HGF IgG alone had no effect on invasion of MMT cells (not shown). Co-cultivation of MMT cells with fibroblasts increased the number of invading cells by a 3.9-fold higher level than that seen without fibroblasts. The number of invading cells in the co-culture was higher than that seen with MMT cells alone in the presence of 5 ng ml$^{-1}$, even though the HGF level in the co-culture was only 1.1 ng ml$^{-1}$ (Figure 9B). This finding suggests that fibroblasts might secrete a factor(s) distinct from HGF and such a factor(s) might stimulate invasion of the cells, in this co-culture system (see below). Importantly, the addition of anti-HGF strongly inhibited invasion of the cells to the level seen in the culture of MMT cells alone. Likewise, the addition of $10^{-7}$ M indomethacin inhibited invasion of MMT cells, but this inhibitory effect was less than that seen with anti-HGF antibody, because the basal HGF production in fibroblasts was retained, even in the presence of indomethacin. Moreover, the addition of $10^{-6}$ M $PGE_2$ to this co-culture in the presence of $10^{-7}$ M indomethacin again stimulated invasion of MMT cells, consistent with the increase in HGF level in this culture condition. Therefore, in this co-culture system, the mutual interaction between MMT cells and fibroblasts was evident: HGF production in fibroblasts is stimulated by $PGE_2$ secreted from MMT cells, while fibroblast-derived HGF in turn strongly stimulates invasion of MMT cells.

Since the enhanced invasion of MMT cells in the co-culture with fibroblasts was inhibited to the basal level by anti-HGF antibody, the above fibroblast-derived factor(s) distinct from HGF is likely to depend on the co-existence of HGF, in stimulating invasion of MMT cells. Although we have yet to specify such a fibroblast-derived factor(s), one possible explanation is that fibroblasts might produce pro-matrix metalloproteinases (pro-MMPs), and the activation of pro-MMPs might depend on tumour-derived proteinase such as u-PA. HGF strongly stimulated u-PA activity in MMT cells (Figure 8).

### DISCUSSION

HGF is a mesenchymal- or stromal-derived factor which elicits mitogenic, motogenic and morphogenic responses in a wide variety of cells (Zarnegar and Michalopoulos, 1995; Matsumoto and Nakamura, 1997). During development of the mammary gland, HGF is involved in branching duct formation in mammary gland epithelial cells (Niranjan et al, 1995; Soriano et al, 1995; Yang et al, 1995). A fibroblast-derived mitogen for mammary gland epithelial cells was identified to be HGF (Sasaki et al, 1994), indicating that HGF affects growth of mammary epithelial cells as a stromal-derived paracrine factor. It is therefore conceivable that HGF functions as a mediator in tumour–stromal interactions in breast cancer, leading to the acquisition of malignant phenotypes in breast cancer cells. In the present study, we found that HGF potently stimulates migration and invasion of MMT mouse mammary carcinoma cells, as well as stimulating their growth. Of importance in our study is the finding that there is a mutual interaction between MMT carcinoma cells and fibroblasts, as mediated by MMT-derived $PGE_2$ and fibroblast-derived HGF. MMT-derived $PGE_2$ up-regulates HGF production in fibroblasts, while fibroblast-derived HGF stimulates growth and invasion of MMT cells. There have been reports that $PGE_2$ levels are elevated in malignant human breast tumours and murine mammary tumours (Rolland et al, 1980; Karmali et al, 1983; Bennett et al, 1989). In-vitro studies using tissue explants or human breast tumour cells in primary culture also revealed higher $PGE_2$ production by tumour cells from malignant tissue than that seen in cells from benign tumour and normal tissues (Watson and Chuah, 1992). Together with these results, one possible mechanism is that breast carcinoma cells secrete $PGE_2$, the elevated $PGE_2$ stimulates HGF production in stromal fibroblasts, and fibroblast-derived HGF induces invasive growth of breast carcinoma cells. We also found that HGF strongly increased u-PA activity in MMT cells. Since involvement of u-PA-induction in tumour invasion has been demonstrated in various cancer cells (Vassalli and Pepper, 1994) and induction of u-PA by HGF correlates with in vitro invasiveness and in vivo metastatic potential (Jeffers et al, 1996), increase in u-PA activity by HGF could possibly be one critical event, leading to increased invasive potential in MMT cells.

Previous studies noted that various types of cancer cells secrete stimulatory factors for production of HGF in distinct types of fibroblasts and such tumour-derived HGF-inducers were identified to be polypeptide cytokines and growth factors such as IL-1α, IL-1β, bFGF and PDGF (Matsumoto et al, 1996; Nakamura et al, 1997). It should be emphasized that we here have newly identified $PGE_2$ to be a non-polypeptide HGF-inducer derived from tumour cells. Previous studies indicated that some prostaglandins ($PGE_1$, $PGE_2$ and $PGI_2$ analogue) stimulate HGF production by activating gene expression of HGF rather than by regulating post-transcriptional processes (Matsumoto et al, 1995). On the other hand expression of HGF gene is up-regulated by at least two distinct signalling pathways: A-kinase-mediated and C-kinase-mediated pathways (Matsunaga et al, 1994; Matsumoto et al, 1995). Although we did not investigate the pathway through which $PGE_2$ exhibits HGF-inducing activity, the A-kinase-mediated pathway is likely to be responsible, for the following reasons: (1) $PGE_2$ stimulates both cAMP levels and HGF production through the EP2/EP4 prostaglandin receptor which stimulates adenylate cyclase (Takahashi et al, 1996); (2) stimulatory effect on HGF production in fibroblasts is additive/synergistic between $PGE_2$ and an activator of C-kinase, but not additive between $PGE_2$ and a cAMP analogue which activates A-kinase (Matsumoto et al, 1995).

Most mammary carcinoma cells and many types of other carcinoma cells do not produce HGF in vitro (Jiang et al, 1993; Byers et al, 1994; Rosen et al, 1994; Nakamura et al, 1997; our unpublished data), whereas many different types of fibroblasts, including mammary fibroblasts produce moderate to high levels of HGF (Stoker et al, 1987; Seslar et al, 1993; Rosen et al, 1994). Thus, stromal cells seem to be a major source of HGF within tumour tissues. On the other hand, there are data that HGF protein and mRNA are expressed in mammary carcinoma cells (Wang et al,

 © 1999 Cancer Research Campaign

1994; Rahimi et al, 1996; Tuck et al, 1996; Jin et al, 1997*a*), as well as in stromal cells. The expression of HGF is elevated in mammary carcinomas, in comparison with findings in benign hyperplasia (Jin et al, 1997*a*; Tuck et al, 1996), and HGF levels in breast cancer tissue are a strong predictor of a recurrence in breast cancer patients (Yamashita et al, 1994; Nagy et al, 1996; Yao et al, 1996). The lack of potential of cultured mammary carcinoma cells to produce HGF may possibly reflect the absence of a stimulatory cofactor required for HGF production in tumour tissues, or a characteristic change of the cells due to consequences of serial cultivation. Although this issue remains to be addressed, the source of HGF in malignant mammary carcinomas may be extended to tumour components, as well as stromal components in more progressed breast cancers. It seems likely that increased $PGE_2$ production in malignant breast cancer cells leads to an increased HGF production in the tumour microenvironment, at least in stromal cells in close proximity to tumour cells.

A number of studies indicate that compounds known as non-steroidal anti-inflammatory drugs (NSAIDs) reduce the incidence of cancers, including colon, bladder, lung and breast cancers (Marnett, 1995; Rosenberg, 1995). Although NSAIDs inhibit cyclooxygenase which catalyses the biosynthesis of $PGH_2$, precursors for prostanoids such as prostaglandins, the mechanisms by which inhibition of PG synthesis contribute to decreased carcinogenesis in colon and some other tissues have yet to be defined. In addition to breast cancer, studies have shown that colon cancer tissues produce a large amount of $PGE_2$ (Narisawa et al, 1990). Moreover, our very recent study showed that a potent stimulatory effect of IL-1 on HGF production in fibroblasts was mediated via $PGE_2$ production and was almost completely abrogated by indomethacin (unpublished data). IL-1 (IL-1$\alpha$ and IL-1$\beta$) are HGF-inducers derived from various tumour cell lines (Matsumoto et al, 1996; Nakamura et al, 1997), and the elevated expression of IL-1 in various tumour tissues has been noted (Colasante et al, 1997; Jin et al, 1997*b*). These results provide insight into one possible mechanism of how the inhibition of PG synthesis by NSAIDs is involved in reduced carcinogenesis. Active PG production in stromal cells as well as in tumour cells in a tumour microenvironment stimulates HGF production, an event which may lead to acquisition of invasive growth potential in cancer cells, and to an increased likelihood of development of a malignant tumour. This hypothesis is now being tested using colon cancer cells.

The neoplastic cells of breast carcinomas are often embedded in stromal tissues, an event which may be important to control their growth, invasion and metastasis. Indeed, stromal cells influence the growth of normal mammary epithelial cells as well as epithelial carcinogenesis in the mammary gland (Tremblay, 1979; van den Hoof, 1988; Lippman and Dickson, 1990; Sakakura, 1991). Our results, at least in part, explain the pathological significance of elevated levels of $PGE_2$ and HGF in malignant cancer tissues, and a possible mechanism for tumour–stromal interaction, as mediated by $PGE_2$ and HGF, which would confer invasive growth potential in breast cancer. If our thesis is tenable, the disruption of such mutual interactions between carcinoma cells and stromal fibroblasts, as mediated by $PGE_2$ and HGF, may possibly be a unique therapeutic strategy toward prevention of invasion and metastasis of breast cancer.

### ACKNOWLEDGEMENTS

We are grateful to M Ohara for useful comments and language assistance and to Dr K Nishikawa (Kanazawa Medical College) for kindly providing a monoclonal antibody against bFGF. This study was supported by a Research Grant for Science and Cancer from the Ministry of Education, Science, Sports, and Culture of Japan, a Research Grant from the Ministry of Welfare of Japan, and grants from Sagawa Cancer Research Foundation, the Ryoichi Naito Foundation for Medical Research, Haraguchi Memorial Foundation for Cancer Research and Tanabe Medical Science Foundation.

### REFERENCES

Bellusci S, Moens G, Gaudino G, Comoglio PM, Nakamura T, Thiery JP and Jouanneau J (1994) Creation of hepatocyte growth factor/scatter factor autocrine loop in carcinoma cells induces invasive properties associated with increased tumorigenicity. *Oncogene* **9**: 1091–1099

Bennett A, Stamford IF, Berstock DA, Dische F, Singh L and A'Hern RP (1989) Breast cancer, prostaglandins and patient survival. *Br J Cancer* **59**: 268–275

Birchmeier C and Birchmeier W (1993) Molecular aspects of mesenchymal–epithelial interactions. *Annu Rev Cell Biol* **9**: 511–540

Bladt F, Riethmacher D, Isenmann S, Aguzzi A and Birchmeier C (1995) Essential role for the c-met receptor in the migration of myogenic precursor cells into the limb bud. *Nature* **376**: 768–771

Bussolino F, Di Renzo MF, Ziche M, Bocchietto E, Olivero M, Naldini L, Gaudino G, Tamagnone L, Coffer A and Comoglio PM (1992) Hepatocyte growth factor is a potent angiogenic factor which stimulates endothelial cell motility and growth. *J Cell Biol* **119**: 629–641

Byers S, Park M, Sommers C and Seslar S (1994) Breast carcinoma: a collective disorder. *Breast Cancer Res Treat* **31**: 203–215

Camps JL, Chang S, Hsu TC, Freeman MR, Hong S, Zhau HE, von Eschenbach AC and Chung LW (1990) Fibroblast-mediated acceleration of human epithelial tumor growth in vivo. *Proc Natl Acad Sci USA* **87**: 75–79

Colasante A, Mascetra N, Brunetti M, Lattanzio G, Diodoro M, Caltagirone S, Musiani P and Aiello FB (1997) Transforming growth factor β1, interleukin-8 and interleukin-1, in non-small-cell lung tumors. *Am J Respir Crit Care Med* **156**: 968–973

Furlong RA, Takehara T, Taylor WG, Nakamura T and Rubin JS (1991) Comparison of biological and immunochemical properties indicates that scatter factor and hepatocyte growth factor are indistinguishable. *J Cell Sci* **100**: 173–177

Grant DS, Kleinman HK, Goldberg ID, Bhargava MM, Nickoloff BJ, Kinsella JL, Polverini P and Rosen EM (1993) Scatter factor induces blood vessel formation in vivo. *Proc Natl Acad Sci USA* **90**: 1937–1941

Grey AM, Schor AM, Rushton G, Ellis I and Schor SL (1989) Purification of the migration stimulating factor produced by fetal and breast cancer patient fibroblasts. *Proc Natl Acad Sci USA* **86**: 2438–2442

Grobstein C (1967) Mechanisms of organogenetic tissue interactions. *Natl Cancer Inst Monogr* **26**: 279–299

Jeffers M, Rong S and Vande Woude GF (1996) Enhanced tumorigenicity and invasion-metastasis by hepatocyte growth factor/scatter factor-met signalling in human cells concomitant with induction of the urokinase proteolysis network. *Mol Cell Biol* **16**: 1115–1125

Jiang WG, Lloyds D, Puntis MC, Nakamura T and Hallett MB (1993) Regulation of spreading and growth of colon cancer cells by hepatocyte growth factor. *Clin Exp Metastasis* **11**: 235–242

Jin L, Fuchs A, Schnitt SJ, Yao Y, Joseph A, Lamszus K, Park M, Goldberg ID and Rosen EM (1997*a*) Expression of scatter factor and c-met receptor in benign and malignant breast tissue. *Cancer* **79**: 749–760

Jin L, Yuan RQ, Fuchs A, Yao Y, Joseph A, Schwall R, Schnitt SJ, Guida A, Hastings HM, Andres J, Turkel G, Polverini PJ, Goldberg D, Rosen EM (1997*b*) Expression of interleukin-1β in human breast carcinoma. *Cancer* **80**: 421–434

Karmali RA, Welt S, Thaler HT and Lefevre F (1983) Prostaglandins in breast cancer: relationship to disease stage and hormone status. *Br J Cancer* **48**: 689–696

Konishi T, Takehara T, Tsuji T, Ohsato K, Matsumoto K and Nakamura T (1991) Scatter factor from human embryonic lung fibroblasts is probably identical to hepatocyte growth factor. *Biochem Biophys Res Commun* **180**: 765–773

Lamszus K, Jin L, Fuchs A, Shi E, Chowdhury S, Yao Y, Polverni PJ, Laterra J, Goldberg ID and Rosen EM (1997) Scatter factor stimulates tumor growth and angiogenesis in human breast cancers in the mammary fat pads of nude mice. *Lab Invest* **76**: 339–353

© 1999 Cancer Research Campaign

Lippman ME and Dickson RB (1990) Growth control of normal and malignant breast epithelium. *Prog Clin Biol Res* **354A**: 147–178

Marnett LJ (1995) Aspirin and related nonsteroidal anti-inflammatory drugs as chemopreventive agents against colon cancer. *Prevent Med* **24**: 103–106

Matsumoto K, Matsumoto K, Nakamura T and Kramer RH (1994) Hepatocyte growth factor/scatter factor induces tyrosine phosphorylation of focal adhesion kinase (p125 FAK) and promotes migration and invasion by oral squamous cell carcinoma cells. *J Biol Chem* **269**: 31807–31813

Matsumoto K, Okazaki H and Nakamura T (1995) Novel function of prostaglandins as inducers of gene expression of HGF and putative mediators of tissue regeneration. *J Biochem (Tokyo)* **117**: 458–464

Matsumoto K, Date K, Shimura H and Nakamura T (1996) Acquisition of invasive phenotype in gallbladder cancer cells via mutual interaction of stromal fibroblasts and cancer cells as mediated by hepatocyte growth factor. *Jpn J Cancer Res* **87**: 702–710

Matsumoto K and Nakamura T (1997) Hepatocyte growth factor (HGF) as a tissue organizer for organogenesis and regeneration. *Biochem Biophys Res Commun* **239**: 639–644

Matsunaga T, Gohda E, Takebe T, Wu YL, Iwao M, Kataoka H and Yamamoto I (1994) Expression of hepatocyte growth factor is up-regulated through activation of a cAMP-mediated pathway. *Exp Cell Res* **210**: 326–335

Matsuzaki K, Yoshitake Y, Matuo Y, Sasaki H and Nishikawa K (1989) Monoclonal antibodies against heparin-binding growth factor II/basic fibroblast growth factor that block its biological activity: invalidity of the antibodies for tumor angiogenesis. *Proc Natl Acad Sci USA* **86**: 9911–9915

Montesano R, Matsumoto K, Nakamura T and Orci L (1991) Identification of a fibroblast-derived epithelial morphogen as hepatocyte growth factor. *Cell* **67**: 901–908

Nagy J, Curry GW, Hillan KJ, McKay IC, Mallon E, Purushotham AD and George WD (1996) Hepatocyte growth factor/scatter factor expression and c-met in primary breast cancer. *Surg Oncol* **5**: 15–21

Nakamura T, Nawa K and Ichihara A (1984) Partial purification and characterization of hepatocyte growth factor from serum of hepatectomized rats. *Biochem Biophys Res Commun* **122**: 1450–1459

Nakamura T, Nishizawa T, Hagiya M, Seki T, Shimonishi M, Sugimura A, Tashiro K and Shimizu S (1989) Molecular cloning and expression of human hepatocyte growth factor. *Nature* **342**: 440–443

Nakamura T, Matsumoto K, Kiritoshi A, Tano Y and Nakamura T (1997) Induction of hepatocyte growth factor in fibroblasts by tumor–derived factors affects invasive growth of tumour cells: in vitro analysis of tumor-stromal interactions. *Cancer Res* **57**: 3305–3313

Narisawa T, Kusaka H, Yamazaki Y, Takahashi M, Koyama H, Koyama K, Fukuda Y and Wakizaka A (1990) Relationship between blood plasma prostaglandin $E_2$ and liver and lung metastases in colorectal cancer. *Dis Colon Rectum* **33**: 840–845

Niranjan B, Buluwela L, Yant J, Perusinghe N, Atherton-A, Phippard D, Dale T, Gusterson B and Kamalati T (1995) HGF/SF: a potent cytokine for mammary growth, morphogenesis and development. *Development* **121**: 2897–2908

Ohmichi H, Koshimizu U, Matsumoto K and Nakamura T (1998) Hepatocyte growth factor (HGF) acts as a mesenchyme-derived morphogenic factor during fetal lung development. *Development* **125**: 1315–1324

Picard O, Rolland Y and Poupon MF (1986) Fibroblast-dependent tumorigenicity of cells in nude mice: implication for implantation of metastases. *Cancer Res* **46**: 3290–3294

Rahimi N, Tremblay E, McAdam L, Park M, Schwall R and Elliott B (1996) Identification of a hepatocyte growth factor autocrine loop in a murine mammary carcinoma. *Cell Growth Differ* **7**: 263–270

Rolland PH, Martin PM, Jacquemier J, Rolland AM and Toga M (1980) Prostaglandin in human breast cancer: evidence suggesting that an elevated prostaglandin production is a marker of high metastatic potential for neoplastic cells. *J Natl Cancer Inst* **64**: 1061–1070

Rosen EM, Joseph A, Jin L, Rockwell S, Elias JA, Knesel J, Wines J, McClellan J, Kluger MJ, Goldberg ID and Zitnik R (1994) Regulation of scatter factor production via a soluble inducing factor. *J Cell Biol* **127**: 225–234

Rosenberg L (1995) Nonsteroidal anti-inflammatory drugs and cancer. *Prev Med* **24**: 107–109

Sakakura T (1991) New aspects of stroma-parenchyma relations in mammary gland differentiation. *Int Rev Cytol* **125**: 165–202

Santos OF, Barros EJ, Yang XM, Matsumoto K, Nakamura T, Park M and Nigam SK (1994) Involvement of hepatocyte growth factor in kidney development. *Dev Biol* **163**: 525–529

Sasaki M, Nishio M, Sasaki T and Enami J (1994) Identification of mouse mammary fibroblast-derived mammary growth factor as hepatocyte growth factor. *Biochem Biophys Res Commun* **199**: 772–779

Seki T, Ihara I, Sugimura A, Shimonishi M, Nishizawa T, Asami O, Hagiya M, Nakamura T and Shimizu S (1990) Isolation and expression of cDNA for different forms of hepatocyte growth factor from human leukocyte. *Biochem Biophys Res Commun* **172**: 321–327

Seslar S, Nakamura T and Byers S (1993) Regulation of fibroblast hepatocyte growth factor/scatter factor expression by human breast carcinoma cell lines and peptide growth factors. *Cancer Res* **53**: 1233–1238

Soriano JV, Pepper MS, Nakamura T, Orci L and Montesano R (1995) Hepatocyte growth factor stimulates extensive development of branching duct-like structures by cloned mammary gland epithelial cells. *J Cell Sci* **108**: 413–430

Stoker M, Gherardi E, Perryman M and Gray J (1987) Scatter factor is a fibroblast-derived modulator of epithelial cell mobility. *Nature* **327**: 239–242

Sykes JA, Whitescarver J and Briggs L (1968) Observations on a cell line producing mammary tumor virus. *J Natl Cancer Inst* **41**: 1315–1327

Tabata MJ, Kim K, Liu J, Yamashita K, Matsumura T, Kato J, Iwamoto M, Wakisaka S, Matsumoto K, Nakamura T, Kumegawa M and Kurisu K (1996) Hepatocyte growth factor is involved in the morphogenesis of tooth germ in murine molars. *Development* **122**: 1243–1251

Takahashi M, Ota S, Hata Y, Mikami Y, Azuma N, Nakamura T, Terano A and Omata M (1996) Hepatocyte growth factor as a key to modulate anti-ulcer action of prostaglandins in stomach. *J Clin Invest* **98**: 2604–2611

Tremblay G (1979) Stromal aspects of breast carcinoma. *Exp Mol Pathol* **31**: 248–260

Tuck AB, Park M, Sterns EE, Boag A and Elliott BE (1996) Coexpression of hepatocyte growth factor and receptor (Met) in human breast carcinoma. *Am J Pathol* **148**: 225–232

van den Hooff A (1988) Stromal involvement in malignant growth. *Adv Cancer Res* **50**: 159–196

Vassalli JD, Dayer JM, Wohlwend A and Belin D (1984) Concomitant secretion of prourokinase and of a plasminogen activator-specific inhibitor by cultured human monocytes-macrophages. *J Exp Med* **159**: 1653–1668

Vassalli JD and Pepper MS (1994) Tumour biology. Membrane proteases in focus. *Nature* **370**: 14–15

Wang Y, Selden AC, Morgan N, Stamp GWH and Hodgson HJF (1994) Hepatocyte growth factor/scatter factor expression in human mammary epithelium. *Am J Pathol* **144**: 675–682

Watson J and Chuah SY (1992) Technique for the primary culture of human breast cancer cells and measurement of their prostaglandin secretion. *Clin Sci (Colch)* **83**: 347–352

Weidner KM, Behrens J, Vandekerckhove J and Birchmeier W (1990) Scatter factor: molecular characteristics and effect on the invasiveness of epithelial cells. *J Cell Biol* **111**: 2097–2108

Weidner KM, Arakaki N, Hartmann G, Vandekerckhove J, Weingart S, Rieder H, Fonatsch C, Tsubouchi H, Hishida T, Daikuhara Y and Birchmeier W (1991) Evidence for the identity of human scatter factor and human hepatocyte growth factor. *Proc Natl Acad Sci USA* **88**: 7001–7005

Wernert N (1997) The multiple roles of tumour stroma. *Virchows Arch* **430**: 433–443

Yamashita J, Ogawa M, Yamashita S, Nomura K, Kuramoto M, Saishoji T and Shin S (1994) Immunoreactive hepatocyte growth factor is a strong and independent predictor of recurrence and survival in human breast cancer. *Cancer Res* **54**: 1630–1633

Yang Y, Spitzer E, Meyer D, Sachs M, Niemann C, Hartmann G, Weidner KM ,Birchmeier C and Birchmeier W (1995) Sequential requirement of hepatocyte growth factor and neuregulin in the morphogenesis and differentiation of the mammary gland. *J Cell Biol* **131**: 215–226

Yao Y, Jin L, Fuchs A, Joseph A, Hastings HM, Goldberg ID and Rosen EM (1996) Scatter factor protein levels in human breast cancers: clinicopathological and biological correlations. *Am J Pathol* **149**: 1707–1717

Zarnegar R and Michalopoulos GK (1995) The many faces of hepatocyte growth factor: from hepatopoiesis to hematopoiesis. *J Cell Biol* **129**: 1177–1180

 © 1999 Cancer Research Campaign
